# Standardization of DNA amount for bisulfite conversion for analyzing the methylation status of *LINE-1* in lung cancer

**DOI:** 10.1371/journal.pone.0256254

**Published:** 2021-08-17

**Authors:** Duong Anh Thuy Pham, Son Duc Le, Trang Mai Doan, Phuong Thu Luu, Uyen Quynh Nguyen, Son Van Ho, Lan Thi Thuong Vo

**Affiliations:** 1 Department of Biochemistry, 175 Hospital, Ho Chi Minh City, Vietnam; 2 Faculty of Biology, University of Science, Vietnam National University, Hanoi, Vietnam; 3 Department of Biology, VNU Institute of Microbiology and Biotechnology, Hanoi, Vietnam; University of Perugia, ITALY

## Abstract

Highly methylated Long Interspersed Nucleotide Elements 1 (*LINE-1*) constitute approximately 20% of the human genome, thus serving as a surrogate marker of global genomic DNA methylation. To date, there is still lacking a consensus about the precise location in *LINE-1* promoter and its methylation threshold value, making challenging the use of *LINE-1* methylation as a diagnostic, prognostic markers in cancer. This study reports on a technical standardization of bisulfite-based DNA methylation analysis, which ensures the complete bisulfite conversion of repeated *LINE-1* sequences, thus allowing accurate *LINE-1* methylation value. In addition, the study also indicated the precise location in *LINE-1* promoter of which significant variance in methylation level makes *LINE-1* methylation as a potential diagnostic biomarker for lung cancer. A serial concentration of 5-50-500 ng of DNA from 275 formalin-fixed paraffin-embedded lung tissues were converted by bisulfite; methylation level of two local regions (at nucleotide position 300–368 as *LINE-1*.*1* and 368–460 as *LINE-1*.*2*) in *LINE-1* promoter was measured by real time PCR. The use of 5 ng of genomic DNA but no more allowed to detect *LINE-1* hypomethylation in lung cancer tissue (14.34% versus 16.69% in non-cancerous lung diseases for *LINE-1*.*1*, p < 0.0001, and 30.28% versus 32.35% for *LINE-1*.*2*, p < 0.05). Our study thus highlighted the optimal and primordial concentration less than 5 ng of genomic DNA guarantees the complete *LINE-1* bisulfite conversion, and significant variance in methylation level of the *LINE-1* sequence position from 300 to 368 allowed to discriminate lung cancer from non-cancer samples.

## Introduction

Alteration of CpG methylation status at either genome-wide or gene-specific level has been confirmed as powerful biomarkers for diagnosis, prognosis, and prediction of diseases [1]. Until now, bisulfite treatment, which specifically converts unmethylated cytosine but not methylated ones to uracil residues [2] is exclusively used in many methods and commercial kits for DNA methylation analysis [3]. For instance, profiling methylation level using bisulfite converted DNA has been performed through PCR-based amplification (MethyLight, Methylation-Sensitive High-Resolution Melting), post-PCR sequencing (conventional Sanger sequencing, pyrosequencing, mass spectrometry-based bisulfite sequencing, next generation sequencing) and methylation arrays [3].

The technical weakness using bisulfite conversion is the incomplete conversion of unmethylated cytosines leading to overestimation of methylation level [4, 5]. In addition, inappropriate conversion of 5-methylcytosine (5mC) to thymine could occur when cytosine conversion achieved complete, thus leading to underestimation of methylation level [6]. Therefore, different commercial bisulfite conversion kits applied to various DNA sources have recently been comprehensively evaluated for their bisulfite conversion efficiency [7–9]. In most of these performance evaluations, only one concentration of different target regions that are single copy sequences were chosen as reference thus limiting the in-depth performance assessment, particularly if one wanted to analyze repeated sequences. These latter constitute about 50% of the human genome [10], and their methylation profile can be used to estimate global DNA methylation for biomarker determination [11, 12]. Indeed, 2 µg of DNA input as usually recommended by most of manufacturers for the methylation analysis of single locus, which only have 2 copies per diploid genome, may not be adapted for repeated sequences, which can have up to 10^5^ copies per genome [13]. Moreover, besides target concentrations, it has been reported that sequence complexity, GC content, secondary structure elements and even a given cytosine in a particular sequence can interfere with bisulfite conversion efficiency [6]. In this delicate technical context, there are still lacking milestones guiding the determination of an optimized genomic DNA input that guarantees complete bisulfite conversion of repeated targets, which is critical to their correct methylation measurement. In addition, not all CpG sites within a single promoter region are functionally equivalent in transcriptional regulation; thus, the precise location of clinically relevant methylated CpGs plays an important role in the development of a DNA methylation-based biomarker [14, 15].

The repetitive DNA retrotransposon Long Interspersed Nucleotide Elements 1 (*LINE-1)* consists of around 5x10^5^ copies in a genome with 3000–4000 copies in full length [13]. The first 460 nucleotide base pairs of its 5’-UTR is particularly important region for the effective transcription, and contains an internal promoter including 33 CpG sites, which are heavily methylated in normal somatic cells [16, 17]. Because of its high frequency in the genome (approximately 20%), the *LINE-1* methylation status could reflect the global DNA methylation level of the genome [12]. Methylation heavily occurs at the *LINE-1* promoter in normal tissues, a mechanism whereby *LINE-1* transcriptional expression and transposition are inhibited, consequently contributing to genome stability [16]. Loss of *LINE-1* methylation promotes chromosomal instabilities and tumor development, which is consistently observed in age related diseases and cancers [10]. *LINE-1* methylation status has thus been considered as a significant diagnostic, prognostic and predictive factor in various types of cancer [18–20]. In most of previous studies, *LINE-1* methylation was quantified thanks to the bisulfite conversion of 100 ng to 2 µg of genomic DNA [1, 21]; however, the methylation values varied broadly among studies, even on the same cancer type [22, 23]. Moreover, most studies were based on only a few specific CpG sites while methylation occurred differently at individual sites [24]. This lack of consensus about the precise location in *LINE-1* promoter and its methylation threshold value may be a part of the reasons delaying the translation of *LINE-1* methylation-based biomarker into available clinical test [25].

We have previously validated an Internal Control (IC) system that allows to evaluate the bisulfite conversion efficiency of unmethylated cytosines by quantitative real time PCR [26]. In the present study, based on this IC system, we investigated the bisulfite conversion efficiency of the repeated *LINE-1* sequences. We showed that an amount of 5 ng, instead of 50 ng or 500 ng of DNA, is appropriate to achieve complete bisulfite conversion of the *LINE-1* target, avoiding over-estimation of *LINE-1* methylation level measured by real time PCR. Using 5 ng of DNA extracted from 275 formalin-fixed, paraffin-embedded (FFPE) tissues, we further showed that two regions within the first 460 base pairs portion of *LINE-1* 5’-UTR was significantly hypomethylated in lung cancer as compared to non-cancerous lung diseases, and their hypomethylation levels were different from each other. There was no significant association of *LINE-1* methylation level with cancer stage.

## Material and methods

### Sample collection, genomic DNA isolation and bisulfite conversion

FFPE tissue samples were collected from 171 lung cancer patients and 104 patients suffering from non-cancerous pulmonary diseases (whose classification was examined by pathologists) at the 175 Hospital (Ho Chi Minh City) during 2018–2020. Informed consent was obtained from healthy participants and patients in written form and the study was approved by the Ethics Committee of Vietnam Academy of Science and Technology (03-2020/NCHG-HDDD). Genomic DNAs were extracted from FFPE lung tissues using the QIAamp DNA FFPE Tissue Kit (Qiagen). After DNA quantification using the NanoDrop2000 –Invitrogen device (S1 Table), serial concentrations ranging from 5 ng, 50 ng and 500 ng of genomic DNAs were subjected to bisulfite conversion using the EZ DNA Methylation-Gold kit (Zymo Research). Bisulfite treated DNAs from 50 ng and 500 ng were diluted 500–1000 times before use for real time PCR. One µl of converted DNA was used as the template in 20 µl reactions.

### Primer design

Primer sets for methylation specific PCR method were designed for measuring methylation level of two regions within the 460 bp portion of *LINE-1* 5′ UTR. The first region is located from 300 bp to 368 bp (named *LINE-1*.*1*) and the second from 368 bp to 460 bp (named *LINE-1*.*2*). Specific primers that are complementary to the sense strand of the bisulfite converted *LINE-1* were designed using the Methyl Primer Express Software v1.0. The methylation-dependent-specific PCR (MSP) primers used for profiling *LINE-1* methylation or unmethylation were derived from the CpGs-containing sequence to ensure their specific annealing to the bisulfite treated target. The methylation-independent-specific PCR (MIP) primers used for quantifying bisulfite converted *LINE-1* were designed from CpGs free region. Primer sequences and their positions on the *LINE-1* promoter (X58075), amplicon lengths and qPCR conditions are shown in S1 Fig and S2 Table.

### Quantitative real time PCR assay

In order to verify primer specificity, real time PCR reactions were performed on (1) bisulfite treated; (2) non-treated DNA, and (3) a mixture of the linearized recombinant plasmids pRef-LINE (containing bisulfite converted *LINE-1* sequence), pMe-LINE (containing methylated *LINE-1* sequence) and pUn-LINE (containing unmethylated *LINE-1* sequence). *LINE-1* methylation status was quantified by real time PCR carried out in 20 µl per reaction using one µl of bisulfite converted DNA as template and SsoAdvanced Universal SYBR Green Supermix (Biorad). Real time PCR assays were duplexed for each of the 3 reactions: (1) using MIP primers to quantify bisulfite converted *LINE-1*; (2) and (3) using MSP primers to quantify methylated *LINE-1* and unmethylated *LINE-1*, respectively. Water with no DNA template was included in each PCR reaction as a control for contamination. All qPCR reactions were performed using the 7500 Real time PCR instrument (Applied Biosystems, CA).

### Methylation calculation

Real time PCR used the primers specific to methylated-CpG and unmethylated-CpG in *LINE-1*.*1* and *LINE1*.*2* regions were designed as methylation and unmethylation reactions (S2 Table). Also, qPCR used the reference primer pair which is not dependent on methylation status was designed as reference reaction. Three adequate formulas: (1) 100 × methylated reaction/(unmethylated reaction + methylated reaction) [27], (2) 100 x (methylated reaction/reference reaction) [28], and (3) the classical ΔΔCT approach using a calibrator reference [29, 30], were chosen to calculate methylation level of the *LINE-1*.*1*, and the formula (3) only used to calculate methylation level of the *LINE-1*.*2*. The percentage of methylation was defined as the ratio between methylated molecules and the sum of methylated and unmethylated molecules or the reference molecules [27], which were calculated based on standard curve performed on serial dilutions from 10^6^ to 10 copies of the linearized recombinant plasmid pMe-LINE, pUn-LINE and pRef-LINE, respectively. Three reactions were carried out for each sample: one reaction that used the *LINE-1* MIP primer set to quantify the total *LINE-1* after bisulfite conversion and two reactions using the *LINE-1* MSP primer sets thus quantifying the methylated and unmethylated *LINE-1*. A serial dilution of the linearized recombinant plasmids pRef-LINE, pMe-LINE and pUn-LINE containing bisulfite converted *LINE-1*, methylated *LINE-1* and unmethylated *LINE-1* sequences, respectively were used as a standard for the measurement of *LINE-1* methylation level. The formula (1) does not use a reference value, while in the formulas (2) and (3), the bisulfite converted *LINE-1* sequence estimated through pRef-LINE was set as reference. To calculate *LINE-1* methylation level following ΔΔCT approach as in the formula (3), a calibrator sample with a defined methylation level of 10% was obtained by mixing linearized pRef-LINE and pMe-LINE plasmids with 10 ng of genomic DNA extracted from lymphocytes of healthy donors. The relative amount of methylated *LINE-1* was calculated for each sample as following: ΔΔCT_Sample_ = ΔCT_Sample_−ΔCT_Calibrator_, where ΔCT_Sample_ = CTSample/Methylated LINE-1 –CT_Sample/Ref- LINE-1_ and ΔCT_Calibrator_ = CTCalibrator/Methylated LINE-1 –CT_Calibrator/Ref-LINE-1_. ΔΔCTs were measured in duplicate. Methylation_Sample_ = Methylation_Calibrator_ x 2^ΔΔCTSample^. Samples were excluded from the study when two replicates of the quantification methylation assay showed CT value > 34.

### Statistical analysis

In all boxplots, methylated and unmethylated *LINE-1* were expressed as medians with interquartile values. Comparisons between more than 2 groups on a quantitative value were assessed either by (i) a mixed model with sample as random effect to account for the fact that the same sample was analyzed using 5 ng, 50 ng or 500 ng DNA; (ii) one-way ANOVA test when conditions on normality and homogeneity of variance were met; or (iii) Kruskal-Wallis test. As for posthoc analysis, the Bonferroni adjustment for multiple comparisons was applied to maintain the type I error at 0.05. Comparisons between 2 groups on a quantitative value were assessed using the Student t-test when normality was met, otherwise by the Wilcoxon test. The association of *LINE-1* with tumor stage was investigated using a linear regression model with adjustment on patients’ age and sex. For all statistical analyses, a p-value < 0.05 was considered as significant. All analyses were performed with the STATA program version 12 (https://www.stata.com/) and Graphpad Prism program version 9 (https://www.graphpad.com/ scientific-software/prism/).

## Results

### Validation of primer specificity and amplification efficiency

As false positive result in DNA methylation analysis could be due to mismatches of primers to unspecific targets [31], the specificity of *LINE-1* primers, designed to specifically recognize methylated *LINE-1* promoter in genomic DNA, was tested by qPCR using either bisulfite treated or untreated genomic DNAs alone, or a mixture of untreated genomic DNAs with different recombinant plasmids (pRef-LINE, pMe-LINE and pUn-LINE) as templates. PCR product was amplified only from specific target templates but unamplified from untreated DNAs, ensuring that the accuracy of MSP primers designed for only methylated targets was guaranteed. The specificity of the primer sets used in the study were thus confirmed and presented in S3 Table and S2 Fig.

### Determination of the optimal genomic DNA quantity for *LINE-1* complete bisulfite conversion

**Fig 1:**
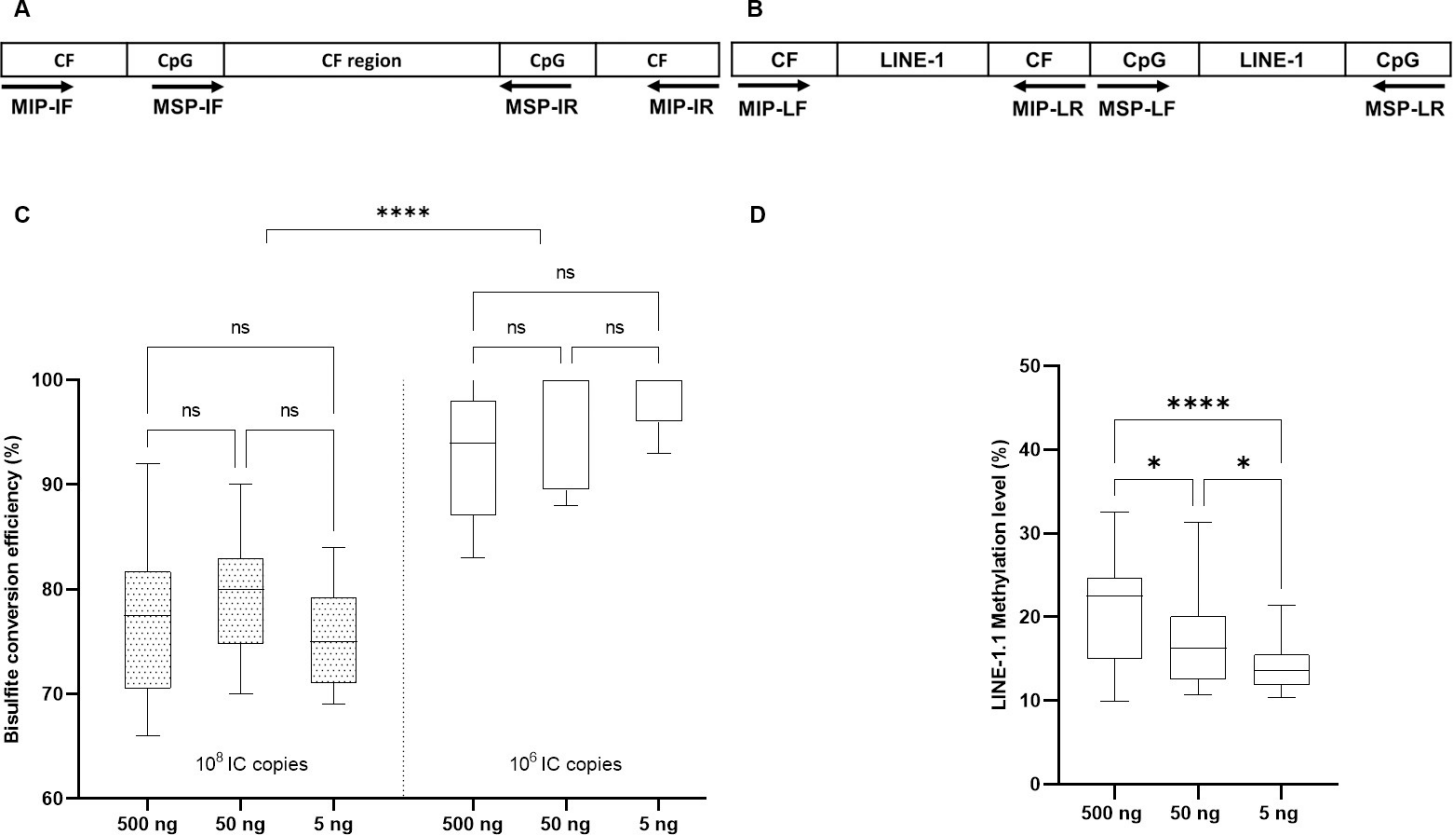
Impact of input genomic DNA quantity on bisulfite conversion efficiency and methylation level. After bisulfite treatment, (A) the internal control (IC) used to monitor bisulfite conversion efficiency, and (B) the *LINE-1*.*1* sequence was amplified thanks to either the MIP primers targeting cytosine free sequences (CF) or the MSP primers targeting CpG sequences, respectively. Bisulfite conversion efficiency of the IC determined from bisulfite-treated samples containing 10^6^ (equivalent ~0.004 ng) or 10^8^ (equivalent ~0.4 ng) IC copies mixed with 500 ng, 50 ng or 5 ng of genomic DNA (C). The comparison in each group (10^6^ or 10^8^ copies) and between two group were done by mixed-effects analysis. *LINE-1*.*1* methylation level calculated from bisulfite-treated samples containing 500 ng, 50 ng or 5 ng of genomic DNA mixed with 10^6^ IC copies (D). One-way ANOVA were used to analyze the data in this experiment.

In order to monitor bisulfite conversion efficiency in DNA methylation assays, we previously validated an artificial internal control (IC) that contained both a cytosine-free (CF) sequence and CpG sequences (Fig 1A). We showed that IC copies number higher than 10^7^ led to incomplete bisulfite conversion [26]. The *LINE-1* gene consists of around 3000–4000 full-length copies in a diploid genome (~ 6 pg), thus ~ 1.5 ng of genomic DNA will contain 10^6^ *LINE-1* full-length copies. Because DNA extracted from FFPE samples is often highly cross-linked, degraded and fragmented, we chose 5 ng of FFPE-extracted DNA (corresponding to ~4x10^6^ *LINE-1* copies) as the minimal amount for bisulfite conversion to ensure sufficient amount of analyzable DNA. To monitor *LINE-1* bisulfite conversion efficiency, either 5, 50 or 500 ng of genomic DNA extracted from 25 FFPE samples was mixed with either 10^6^ (equivalent ~ 0.004 ng) or 10^8^ copies (~ 0.4 ng) of IC and submitted to bisulfite treatment. The converted products were then amplified with the MIP or MSP primers, matching respectively the CF or CpG sequences present in the IC (Fig 1A) and *LINE-1*.*1* (Fig 1B). It is worth noting that the primer set specific to the bisulfite converted *LINE-1*.*1* sequences did not produce any PCR product from bisulfite treated IC sequence, confirming its specificity (results not shown). Bisulfite conversion efficiency of the IC calculated by the ΔΔCT method was incomplete (< 80%) when 10^8^ IC copies were used, while an efficiency of over 90% was observed when using 10^6^ IC copies, regardless of the genomic DNA amount (Fig 1C). Conversion efficiency of IC changed from 75% to 95% when 10^8^ or 10^6^ IC copies were bisulfite treated with 5 ng of genomic DNA (Fig 1C). The direct sequencing of PCR products indicated that all cytosines on the IC were completely converted to thymines when 10^6^ IC copies were mixed with 500 ng, 50 ng and 5 ng genomic DNA while the conversion was incomplete when 10^8^ IC copies were used, either mixed with 500 ng or 5 ng or genomic DNA (S3A–S3C Fig). Moreover, the methylation level of *LINE-1*.*1* significantly varied following the genomic DNA amount used for bisulfite treatment (Fig 1D). As for *LINE-1*.*1* conversion, all *LINE-1*.*1* non CpG cytosines was completely converted using 5 ng genomic DNA but the conversion remained incomplete with higher DNA quantity (S3D–S3F Fig). This result suggested that low conversion efficiency was due to high copy number of the IC and *LINE-1* targets.

**Fig 2:**
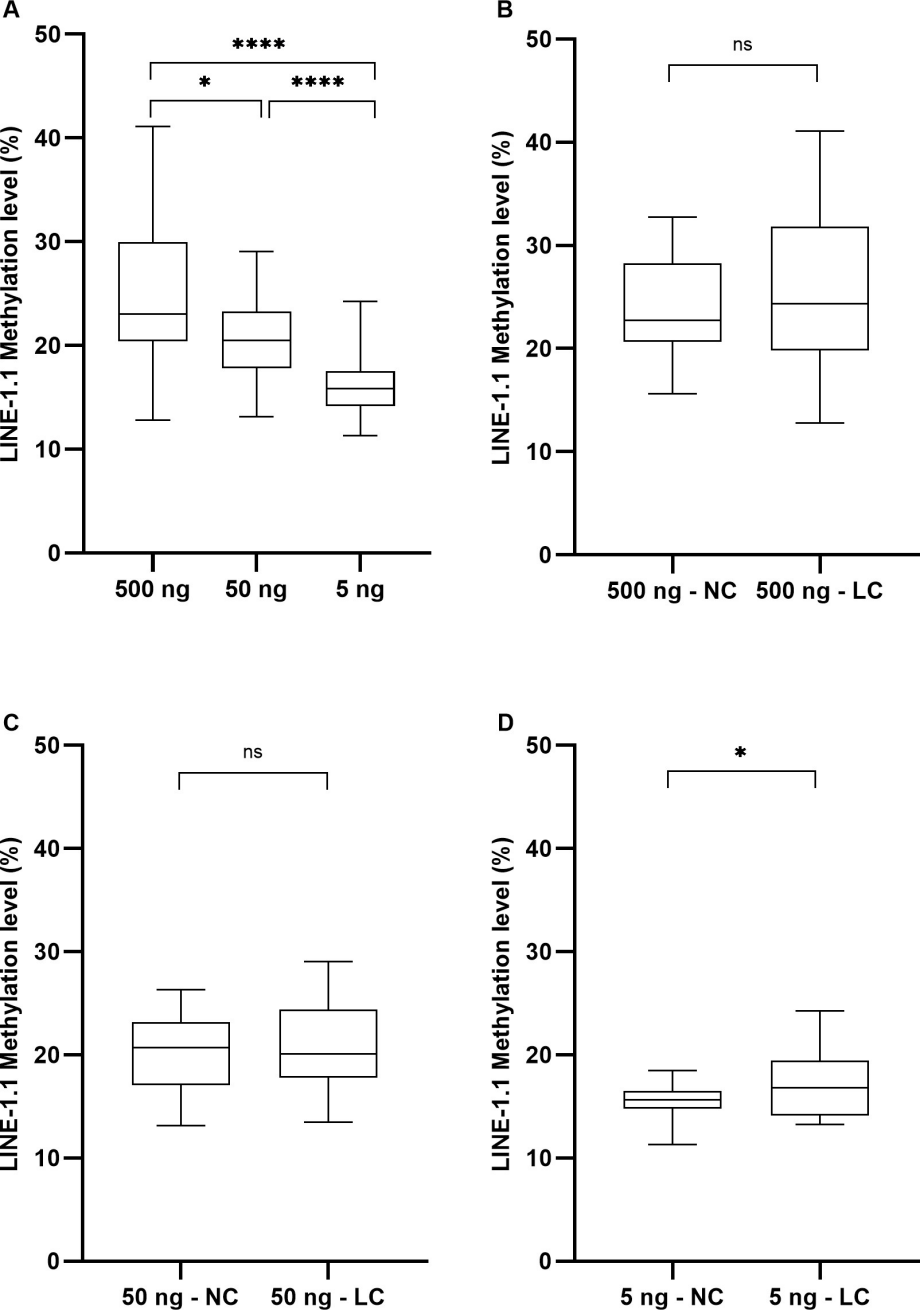
*LINE1* methylation level depends on the quantity of input genomic DNA submitted to bisulfite conversion. *LINE-1*.*1* methylation level determined from 500 ng, 50 ng or 5 ng of genomic DNA extracted from FFPE tissue and submitted to bisulfite conversion, no matter the samples are lung cancer (LC) or non-cancerous lung disease (NC) (A), in LC *versus* NC samples when using 500 ng (B), 50 ng (C) and 5 ng (D) of genomic DNAs. (*) p < 0.05; (**) p < 0.01; (***) p < 0.001; (****) p < 0.0001. One-way ANOVA (A) and Welch’s test (B-D) were used in statistical analysis.

In order to ascertain the impact of DNA amount on *LINE-1* methylation measurement, 60 FFPE samples from lung cancer (30 samples) and non-cancerous lung disease patients (30 samples) were converted by bisulfite, testing 3 DNA quantities, 5 ng– 50 ng– 500 ng for each sample. We observed significant differences in *LINE-1*.*1* methylation level according to DNA quantity (Fig 2A). Using 500 ng or 50 ng genomic DNA, *LINE-1*.*1* methylation values (24.85% and 20.40%, respectively) are significantly higher as compared to 16.22% (p < 0.0001) observed with 5 ng DNA, associated with higher measurement variances, suggesting a proportion of false-positive artifacts probably derived from incomplete bisulfite conversion. Those artifacts may mask the difference in *LINE-1*.*1* methylation between lung cancer and non-cancerous lung diseases, which were not observed when using high quantity of genomic DNA (Fig 2B and 2C) but appeared significant when using 5 ng of input DNA (17.19% versus 15.25%, p < 0.05, respectively) (Fig 2D). Overall, those results indicated that 5 ng of genomic DNA is the optimal DNA quantity ensuring the complete bisulfite conversion of *LINE-1* sequences, thus allowing the discrimination of lung cancer against non-cancerous lung diseases based on *LINE-1* methylation level.

### Selection of the calculation method for quantifying *LINE-1* methylation

**Fig 3:**
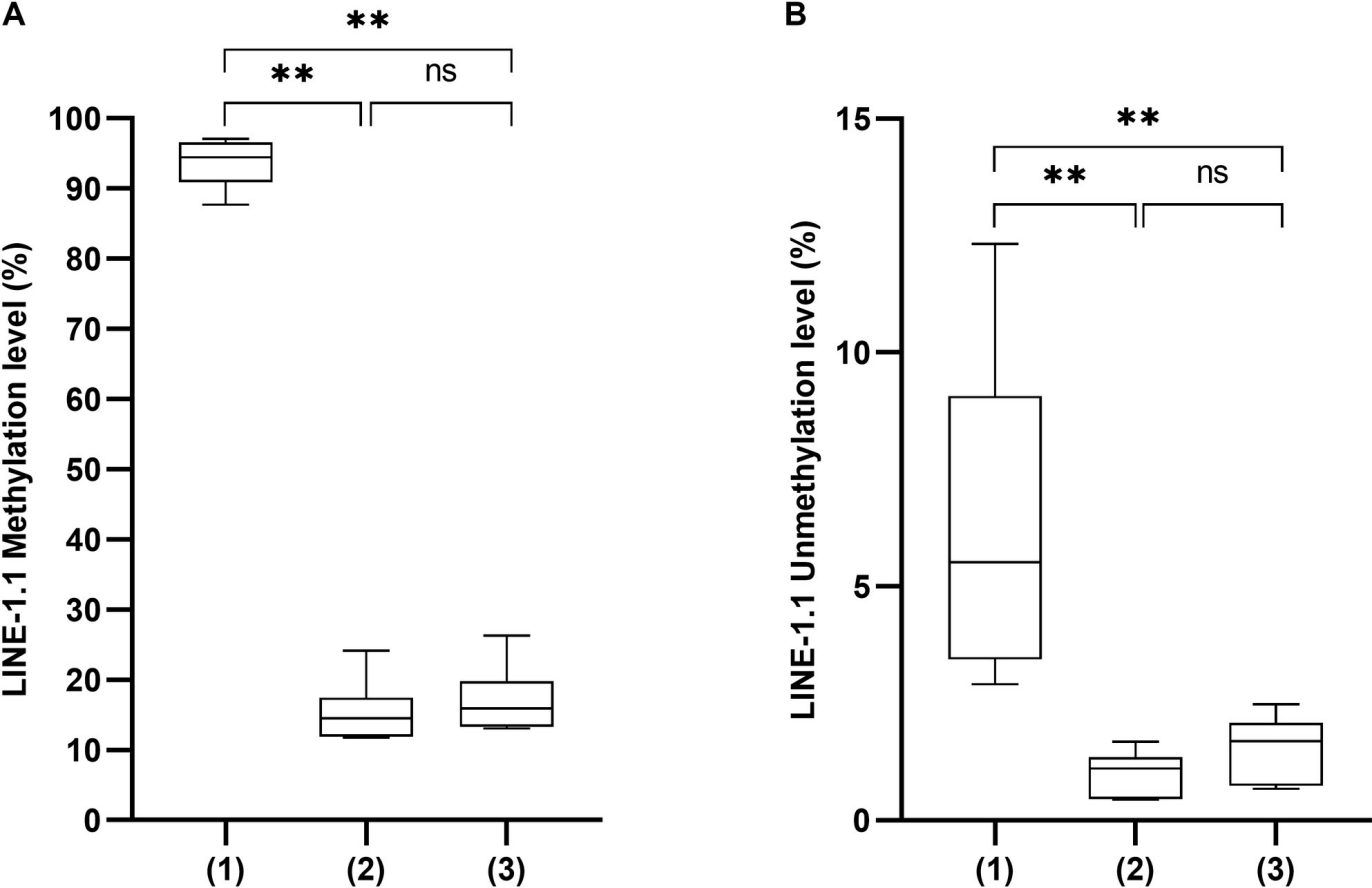
The methylated (A) and unmethylated (B) percentage of *LINE-1*.*1* sequences calculated by three formulas (1), (2) and (3) (see text) using 5 ng of DNA extracted from FFPE samples and bisulfite converted. (*) p < 0.05; (**) p < 0.01; (***) p < 0.001; (****) p < 0.0001. Statistical analysis was done by one-way ANOVA.

Before enlarging *LINE-1* methylation analysis for *LINE-1*.*1* and *LINE-1*.*2* regions on the whole sample set using the optimal 5 ng genomic DNA, we proceeded to the selection of the optimal methylation calculation formula, by comparing the ΔΔCT approach that we had used until now with two other formulas in the literature. We thus compared the methylated *LINE-1* percentage obtained following three formulas: (1) 100 × methylated reaction/(unmethylated reaction + methylated reaction), (2) 100 x (methylated reaction/reference reaction) and (3) the classical ΔΔCT approach using a calibrator reference. Analyses were performed on 10 FFPE samples, using 5 ng of bisulfite converted genomic DNA and three primer sets specific to the unmethylated, methylated and bisulfite converted *LINE-1*, as detailed in the Material and Methods section. With the formula (1), the methylated *LINE-1*.*1* percentage was around 93.7%, significantly much higher than those calculated with the formulas (2) and (3), which showed similar values of 15.4% and 17.0%, respectively (Fig 3A). Moreover, in our hands, the unmethylated *LINE-1*.*1* calculated with the formula (1) showed greater measurement variance compared to those calculated with the formulas (2) and (3) (Fig 3B). Given its feasibility and popularity, the ΔΔCT method was retained for calculating the *LINE-1*.*1* and *LINE-1*.*2* methylation levels in further analysis.

### Analysis of *LINE-1*.*1* and *LINE-1*.*2* methylation levels in patients with lung cancer and non-cancerous lung diseases

**Fig 4:**
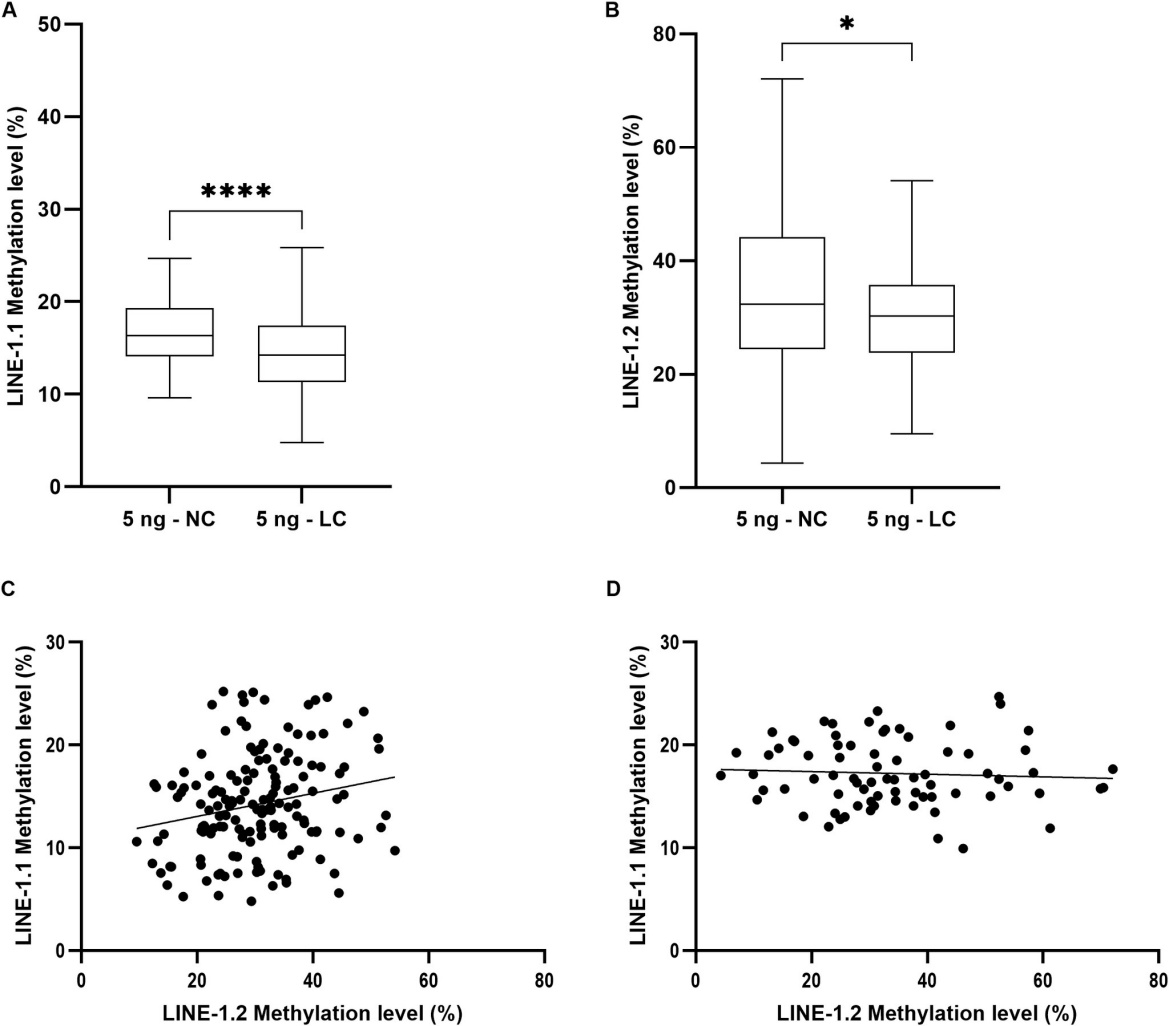
*LINE-1* methylation. Hypomethylation of *LINE-1*.*1* (A) and *LINE-1*.*2* (B) in NC and LC. Linear regression correlation between *LINE-1*.*1* and *LINE-1*.*2* in LC group (C) and NC group (D). Methylation measurement was performed on 5 ng of DNA converted by bisulfite. Welch’s t test was used in (A) and (B) while multiple linear regression was used in (C) and (D). (*) p < 0.05; (**) p < 0.01; (***) p < 0.001; (****) p < 0.0001.

In order to gain insight into a possible diagnostic value of *LINE-1* methylation level in lung cancer, we extended the *LINE-1* methylation analysis for two regions, *LINE-1*.*1* (position 300–368) and *LINE-1*.*2* (position 368–460), respectively on 275 FFPE samples (171 patients with lung cancer *versus* 104 patients with non-cancerous lung diseases), using 5 ng genomic DNA for bisulfite conversion. A significant hypomethylation was observed for both *LINE-1*.*1* and *LINE-1*.*2* (Fig 4A and 4B). As observed on the test sample set (Fig 2D), the fully methylated *LINE-1*.*1* level in lung cancer (14.34%) was significantly lower than that in non-cancerous lung disease samples (16.69%) (p < 0.0001). The methylated *LINE-1*.*2* level in lung cancer patients (30.28%) was significantly lower than in non-cancerous ones (32.35%) (p < 0.05). We also observed a linear regression correlation between *LINE-1*.*1* and *LINE-1*.*2* in malignant samples (with slope value is 0.1124; p < 0.01, and Y intercept value is 10.79; p < 0.0001) (Fig 4C) but not in benign ones (Fig 4D).

### Association of *LINE-1* methylation level with lung cancer pathological characteristics

We further analyzed the association of *LINE-1* methylation level with tumor stage, *EGFR* mutation status, adjusting for patients’ age and gender using multiple linear regression. Among a total of 171 FFPE samples from lung cancer patients analyzed for *LINE-1*.*1* methylation, only 162 had data recorded with age and gender, 110 had *EGFR* mutation status and 90 had tumor stage information. All of samples are non-squamous non-small cell lung cancer (NSCLC) samples, which corresponds to the most frequent lung cancer histologic subtype. Thus, we analyzed those samples and did not include the histological characteristics in the regression model. No statistically significant linear dependence of *LINE-1*.*1* (Table 1A) or *LINE-1*.*2* (Table 1B) on age, cancer stage and *EGFR* mutation status was detected (all regression coefficients had p > 0.05).

**Table 1:** Association of *LINE-1*.*1* (A) and *LINE-1*.*2* (B) methylation status with cancer stage and patients’ demographical characteristics in non-squamous NSCLC analyzed by multiple linear regression.

	Feature	Regression coefficient	95% CI	P value
**A**				
	Age (in years)	0.02979	[-0.1748; 0.2344]	0.7707
	Gender (Male *versus* Female)	-2.093	[-6.199; 2.012]	0.3100
	Tumor stage (Metastatic *versus* Non-metastatic)	4.858	[-1.873; 11.59]	0.1530
	*EGFR* mutation status	-1.157	[-5.560; 3.246]	0.5992
**B**				
	Age (in years)	-0.1749	[-0.5903; 0.2405]	0.3998
	Gender (Male versus Female)	-4.083	[-12.61; 4.440]	0.3388
	Tumor stage (Metastatic versus Non-metastatic)	9.864	[-2.946; 22.67]	0.1275
	*EGFR mutation status*	-6.390	[-15.38; 2.598]	0.1585

## Discussion

To date, bisulfite conversion is indispensable for DNA methylation profiling either on the whole genome or on a particular target [3]. In fact, there are some methods that does not require bisulfite conversion but instead use methylated CpG binding proteins or methylation sensitive endonucleases. However, none of those can focus on specific CpG sites of interest but likely bias results towards CpG-dense regions due to the difference in antibody combination or enzymatic digestion efficiency, respectively [32]. Thus, bisulfite-based methods have been considered as the gold standard in DNA methylation analysis with current challenges consisting in maximizing bisulfite efficiency since incompletely converted DNA templates produce biases and errors in methylation measurement [4, 5]. Most assays evaluating bisulfite conversion efficiency have been performed on single copy sequences with only 2 copies in a genome [7, 8]. As to repetitive sequences that can reach up to 10^5^ copies and representing about 50% of the human genome, no report on their bisulfite conversion efficiency have been described so far.

*LINE-1* is the only autonomously active family in human genome. *LINE-1* mobilizes itself and *ALU* and *SVA* retrotransposons *in trans*, leading great impact on genome structure, gene transcription and various other functional consequences [33]. *LINE-1* expression is strictly controlled through DNA methylation. Over 90% of methylated CpG sites in the human genome occur particularly in the *LINE-1* and *Alu* [34]. Loss of *LINE-1* methylation promotes tumor development and is associated with cancer recurrence [10]. *LINE-1* methylation status has thus been considered as a significant diagnostic, prognostic and predictive factor in various types of cancer [18–20]. However, the standardization of *LINE-1* methylation as a clinical biomarker for cancer and treatment response prediction has been hindered by substantial inconsistencies in *LINE-1* methylation measurement over various studies using bisulfite-based methods. Indeed, *LINE-1* methylation widely ranged from 14.8% to 78.8% in non-small cell lung cancer tissues [35], from 21.3% to 76% in ovarian cancer [36] and from 6% to 94.0% in oropharyngeal squamous cell carcinoma [27]. A disagreement about diagnostic value of *LINE-1* methylation has been debated among studies on the same cancer type [22, 23]. A controversial hypermethylation of *LINE-1* has been also reported to colorectal and cervical cancers [19, 37]. Recently, a multicenter benchmarking study using a wide range of DNA amount (45 ng– 1.6 µg) and comparing the performance of different bisulfite-based DNA methylation assays realized on 32 reference samples in 18 laboratories in 7 different countries showed divergent results of *LINE-1* methylation levels between assays [1]. All these discrepancies could be due to one part by methylation level was measured on different regions of the *LINE-1* promoter [22, 23] and other part by a large range of DNA amount (50 ng– 2 µg) was used for bisulfite treatment [36, 38]. In this context, our study aimed at optimizing genomic DNA input that ensures complete bisulfite conversion for accurately measurement of *LINE-1* methylation and opting for *LINE-1* local region of which methylation level could be a potential diagnosis marker for lung cancer.

To our knowledge, this study is the first pointing out that the input genomic DNA quantity should not exceed 5 ng, much less than what have been used so far. Based on the internal control system for bisulfite conversion efficiency that we have recently validated [26], we demonstrated that *LINE-1* sequences were completely converted when using 5 ng DNA (Fig 1, S3 Fig). Methylation value obtained from such bisulfite converted DNA was 14.34% in lung cancer samples (Fig 4A), much less than what have been previously described for lung cancer (> 70%) [35, 39] but consistent with the global *LINE-1* methylation (~5%) assessed by non-bisulfite approaches [40, 41]. In contrast, when using higher DNA quantities (50 ng– 500 ng), *LINE-1* sequences were incompletely bisulfite converted (Fig 1, S3 Fig), which may be source of artifacts behind higher methylation values (up to 25%) associated with greater measurement variances (Fig 2). More importantly, decreasing the DNA amount down to 5 ng allowed to distinguish lung cancer against non-cancerous lung diseases based on *LINE-1* methylation in FFPE tissue-extracted DNA (Fig 2D, Fig 4), which could not be achieved with higher DNA amount (Fig 2B and 2C). Our observation is in line with the results obtained from various protocols in the literature, which have clearly highlighted the relation between DNA input quantity and skewed *LINE-1* methylation values. For instance, *LINE-1* methylation levels quantified by bisulfite-based pyrosequencing assay using 1 µg or 250 ng of genomic DNA were all different and much higher (>70%) as compared to the *LINE-1* methylation level determined by HPLC as reference (5.2%) [42, 43]. Liu and collegues (2013) have recommended less than 100 ng of DNA for bisulfite sequencing of repetitive elements [21]. Recently, only 30 ng of DNA was recommended to assess *LINE-1* methylation in cervical intraepithelial neoplasia [19]. Our result is in line with the fact that *LINE-1* hypomethylation is predominantly observed in cancer tissues assessed by non-bisulfite approaches using methylation-sensitive endonucleases or Methylation-Specific Multiplex Ligation-dependent Probe Amplification (MS-MLPA) [44, 45]. By directly demonstrating the impact of DNA quantity in *LINE-1* methylation measurement, our findings contributed to standardize bisulfite-based protocols for DNA methylation assay particularly applied to repetitive targets, in order to integrate global DNA methylation as a biomarker in cancer diagnostic and monitoring.

Aberrant methylated CpG hotspots at the 5’ region of single copy gene or repetitive sequences were associated with tumor specificity [15, 24]. The internal region from position 300 to 460 within *LINE-1* 5’UTR was heavily methylated in normal cells [16, 17] and hypomethylated differently in cancer types [15]. Four CpG sites in positions 305 to 331 have been chosen for quantifying methylation variance by commercially available pyrosequencing assay (PyroMark LINE‐1 kit, Qiagen, Hilden, Germany). In MSP method, MSP primers have been designed based on CpG sites in different target regions, which could lead to an inconsistence in *LINE-1* methylation value. Thus, in this study, two MSP primer sets were created for *LINE-1*.*1* and *LINE-1*.*2* regions which contain total 11 CpG sites from base pair 300 to 460 within the *LINE-1* 5’-UTR. *LINE-1*.*1* was less methylated (16.69%) than *LINE-1*.*2* (32.35%) in non-cancerous lung diseases but more hypomethylated (14.34%) than *LINE-1*.*2* (30.28%) in lung cancer (S4 Fig). The specificity of methylation in non-cancerous diseases and hypomethylation in lung cancer between two regions could be explained by potential transcription factor binding sites for *LINE‐1* [17] and epigenetic heterogeneity in cancer [46]. Different variance in *LINE-1*.*1* and *LINE-1*.*2* methylation indicated that target sequence for methylation measurement should be carefully chosen since altered methylation levels could not be equally detectable at all CpG sites. The MSP primers specific to CpGs in *LINE-1*.*1* allowed discriminating significantly lung cancer from non-cancerous lung diseases better than the one in *LINE-1*.*2*. Although *LINE-1*.*1* and *LINE-1*.*2* were hypomethylated in lung cancer, consisting with previous reports on decrease in *LINE-1* methylation in various type of cancer [10, 15], there was no association of their methylation level with age, gender, and cancer stage. These results are in line with former observations determined by non-bisulfite-based approaches [37, 45]. In contrast, the conflict to our result have been shown in some previous studies in which more than 500 ng of DNA was used for bisulfite conversion [11, 47]. A broad range of input DNA amount used for bisulfite treatment in described studies might be the reason that makes the multiple linear regression analysis to be more challenging. Otherwise, highly heterogeneity of tumor cells and differential methylation could be the explanation to the contradictory [48, 49].

A minor attention should be drawn on the method for calculating the methylation level of repetitive elements. Indeed, there are several formulas for absolute or relative calculations, based on the quantitation of unmethylated target or a calibrator reference, respectively. Since the unmethylated *LINE-1* value is low (around 1–6%) but varies according to the calculation method (Fig 3B), the calibrator may be a more convenient reference for *LINE-1* methylation measurement (S5 Fig). It is worth noting that the reference must be chosen from repeat elements to overcome the limited detection of single copy references when using low quantities of input DNA [30]. Standardizing all the steps in methylation-based biomarker assay protocols will promote the consolidation and performance of methylation-based marker [25].

Our study has some limitations. Firstly, the DNA concentration is calculated based on the absorbance at 260 nm using UV-VIS, which could be less sensitive and less specific than that calculated using fluorescence dye since the latter distinguishes DNA and RNA molecules. Moreover, as this study is a primary validation of the optimal DNA amount for accurate *LINE-1* methylation analysis, we have set 5 ng of genomic DNA as the minimal amount ensuring analyzable material, having accounted for DNA degradation. Since this amount is still 10 times higher than the 0.5 ng theoretically calculated based on the copy number of the *LINE-1* in a genome, in future studies, amounts less than 5 ng of genomic DNA from various sources (fresh tissue, blood, urine, bronchial aspirate or washing during bronchoscopy…) should be more finely investigated. In addition, inappropriate conversion of 5mC to thymine should be also investigated since it could take place when cytosine conversion achieved complete [6], thus leading to underestimation of *LINE-1* methylation level. Particularly, the abundance of the *LINE-1* copies in circulating cell free DNA (ccfDNA) was more than that in the genome [50]. Thus, despite the low DNA concentration isolated from noninvasive liquid biopsy samples, an appropriate ccfDNA amounts used for measurement of *LINE-1* methylation value should be evaluated in respect to making it as an attractive methylation-based cancer biomarker.

## Conclusion

To summarize, this study has proposed a technical standardization of bisulfite-based methylation analysis particularly applied to repetitive targets, and precise region in the *LINE-1* promoter of which variance in DNA methylation level has diagnostic potency in lung cancer. We have showed that an input DNA amount no more than 5 ng was optimal to ensure the complete bisulfite conversion of repeated *LINE-1* sequences, which allows to measure methylation level accurately. In such a way, methylation in *LINE-1* sequence from position 300 to 480 could be conferred as a biomarker to lung cancer. These encouraging results prompt us to quantitatively assess *LINE-1* methylation in noninvasive liquid biopsy samples, in the common effort to foster the use of global DNA methylation analysis in biomarker development and clinical applications.

## Supporting information

S1 Table Quantification of DNA extracted from lung cancer and non-cancerous FFPE samples. (XLSX)

S2 Table Primer sets and quantitative real time PCR conditions for measurement of *LINE-1* methylation. (DOCX)

S3 Table Specificity of primer sets used for the amplification the methylated *LINE-1*.*1*. gDNA: Non converted genomic DNA (10 ng/reaction); pRef-LINE, pMe-LINE-1.1 and pUn-LINE-1.1: Linearized recombinant plasmids (4 pg equivalent to 10^6^ copies); undetermined: Ct>36. Average CT values were calculated from 5 repeated reactions.
(DOCX)

S1 Fig Nucleotide sequence of the *LINE-1* promoter (X58075) and position of MIP (Ref-F/R) and MSP (Me1-Line-F/R and Me2-Line-F/R) primers used for methylation analysis of *LINE-1*.*1* and *LINE-1*.*2* regions. (DOCX)

S2 Fig Plasmid pMe-LINE1.1 was mixed with plasmid pUnMe-LINE1 in different ratios. Standard curves of pMe-LINE1.1 of each mix were built by using simple linear regression analysis. The different between the curves (Slope and Y-intercept) is not significant (p = 0.1225 and p = 0.9085 respectively). The pooled slope equals -3.409 and the pooled Y-intercept equals 36.19.
(DOCX)

S3 Fig IC and *LINE-1* sequencing results. Direct sequencing of the IC amplified from bisulfite-treated samples containing 10^6^ IC copies mixed with 500 ng of DNA (A) or 10^8^ IC copies mixed with either 500 ng (B) or 5 ng (C) of genomic DNA. Direct sequencing of the *LINE-1* sequence amplified from bisulfite-treated samples containing 10^6^ IC copies and 5 ng (D), 50 ng (E) and 500 ng (F) of genomic DNA. Unconverted cytosines were indicated by arrows.
(DOCX)

S4 Fig *LINE-1* methylation. Methylation level between *LINE-1*.*1* and *LINE-1*.*2* in non-cancerous lung diseases-NC (A) and in lung cancer-LC (B). Methylation measurement was performed on 5 ng of DNA converted by bisulfite. (*) p < 0.05; (**) p < 0.01; (***) p < 0.001; (****) p < 0.0001. Welch’t test were used for analysis.
(DOCX)

S5 Fig Validation of the reference for *LINE-1* methylation measurement. (A) Invariability of ΔCT value analysed by ANOVA statistical method when using qPCR templates as following: a serial concentration of the pRef-LINE-1 (read line) and a serial concentration of the pMe-LINE-1.1 (blue line). (B) Validation of ΔΔCT method. The amplification efficiency of the bisulfite converted *LINE-1*.*1* and methylated *LINE-1*.*1* targets was examined using qPCR. A 10% methylation LINE-1 calibrator was made by mixing pMe-LINE-1.1 with pRef-LINE-1 in ratio 1:10 and the serial dilution of the mixture was made, then amplified by methylation independent and methylation dependent primer sets, respectively. The ΔCT (CT_Me-LINE-1.1_-CT_Ref-LINE-1.1_) was calculated for each dilution. The data was fit using least-squares linear regression analysis (N = 9).
(DOCX)
