# Supplementary material for: Standardization of DNA amount for bisulfite conversion for analyzing the methylation status of LINE-1 in lung cancer
Source: PLoS One. 2021 Aug 17;16(8):e0256254. doi: 10.1371/journal.pone.0256254 (PMC8370637; doi:10.1371/journal.pone.0256254)
Supplement: S2 Table — (DOCX) [file pone.0256254.s002.docx]

**S2 Table. Primer sets and quantitative real time PCR conditions for measurement of *LINE-1* methylation**

| **MIP primers** | **MSP primers** | **Sequence (5’–3’)** | **Amplicon size (bp)** | **qPCR conditions** |
| --- | --- | --- | --- | --- |
| Ref-F |  | GT AAG GGG TTA GGG AGT TTT T | 87 | 95°C 5 min, 40 cycles of (95°C 10 sec, 63°C 30 sec, 72°C 30 sec), 72°C 5 min. |
| Ref-R |  | CAATATTCGGGTGGGAGTGACC |  |  |
|  | Me1-Line-F | CGG TTT AAG AAA CGG CGT ATT AC | 82 |  |
|  | Me1-Line-R | ACA ATC AAC GAA ATT CCG TAA ACG |  |  |
|  | Un1-Line-F | AGTCGTATTCGTAGACGTTTTTC | 90 |  |
|  | Un1-Line-R | AAACGCCTATACTCGTACG |  |  |
|  | Me2-Line-F | GGGTTTTACGTTTACGGAATTTC | 81 |  |
|  | Me2-Line-R | CCCCCAACCTCGTTA |  |  |
|  | Un2-Line-F | AGAGGGTTTTATGTTTATGGAATTTT | 92 |  |
|  | Un2-Line-R | CCTCCCCCAACCTCATTA |  |  |
