# Supplementary material for: Standardization of DNA amount for bisulfite conversion for analyzing the methylation status of LINE-1 in lung cancer
Source: PLoS One. 2021 Aug 17;16(8):e0256254. doi: 10.1371/journal.pone.0256254 (PMC8370637; doi:10.1371/journal.pone.0256254)
Supplement: S3 Table — gDNA: Non converted genomic DNA (10 ng/reaction); pRef-LINE, pMe-LINE-1.1 and pUn-LINE-1.1: Linearized recombinant plasmids (4 pg equivalent to 106 copies); undetermined: Ct>36. Average CT values were calculated from 5 repeated reactions. (DOCX) [file pone.0256254.s003.docx]

**S3 Table. Specificity of primer sets used for the amplification the methylated *LINE-1.1*.** gDNA: Non converted genomic DNA (10 ng/reaction); pRef-LINE, pMe-LINE-1.1 and pUn-LINE-1.1: Linearized recombinant plasmids (4 pg equivalent to 10^6^ copies); undetermined: Ct>36. Average CT values were calculated from 5 repeated reactions

| Primer pair | CT values of Real time PCR amplified from the templates | | | | |
| --- | --- | --- | --- | --- | --- |
|  | gDNA | gDNA+pMe-LINE-1.1 | gDNA+pMe-LINE-1.1+pUn-LINE-1.1 | gDNA+pRef-LINE | gDNA+pRef-LINE + pMe-LINE-1.1 |
| Me1-Line-F/Me1-Line-R | undetermined | 14.78 ± 0.14 | 14.80 ± 0.21 | undetermined | 14.80 ± 0.24 |
| Un1-Line-F/Un1-Line R | undetermined | undetermined | 13.53 ± 0.37 | undetermined | undetermined |
| Ref-F/Ref-R | undetermined | undetermined | undetermined | 15.76 ± 0.12 | 15.63 ± 0.16 |
