# Supplementary material for: Standardization of DNA amount for bisulfite conversion for analyzing the methylation status of LINE-1 in lung cancer
Source: PLoS One. 2021 Aug 17;16(8):e0256254. doi: 10.1371/journal.pone.0256254 (PMC8370637; doi:10.1371/journal.pone.0256254)
Supplement: S1 Fig — (DOCX) [file pone.0256254.s004.docx]

**
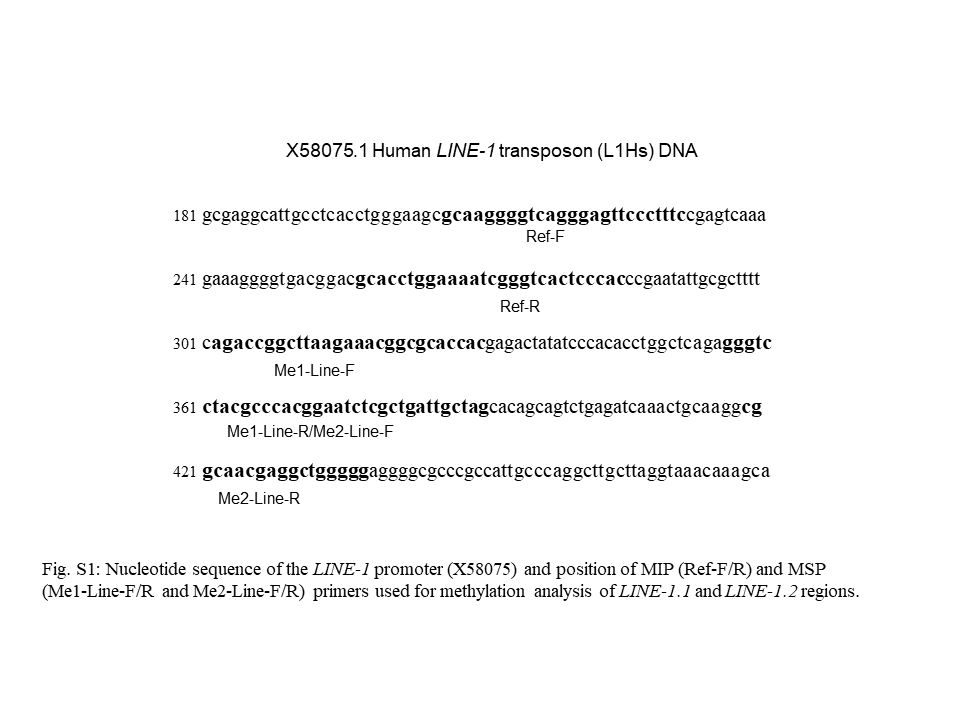
**

**S1 Fig.** **Nucleotide sequence of the *LINE-1* promoter (X58075) and position of MIP (Ref-F/R) and MSP (Me1-Line-F/R and Me2-Line-F/R) primers used for methylation analysis of *LINE-1.1* and *LINE-1.2* regions.**
