# Supplementary material for: Standardization of DNA amount for bisulfite conversion for analyzing the methylation status of LINE-1 in lung cancer
Source: PLoS One. 2021 Aug 17;16(8):e0256254. doi: 10.1371/journal.pone.0256254 (PMC8370637; doi:10.1371/journal.pone.0256254)
Supplement: S2 Fig — Standard curves of pMe-LINE1.1 of each mix were built by using simple linear regression analysis. The different between the curves (Slope and Y-intercept) is not significant (p = 0.1225 and p = 0.9085 respectively). The pooled slope equals -3.409 and the pooled Y-intercept equals 36.19. (DOCX) [file pone.0256254.s005.docx]

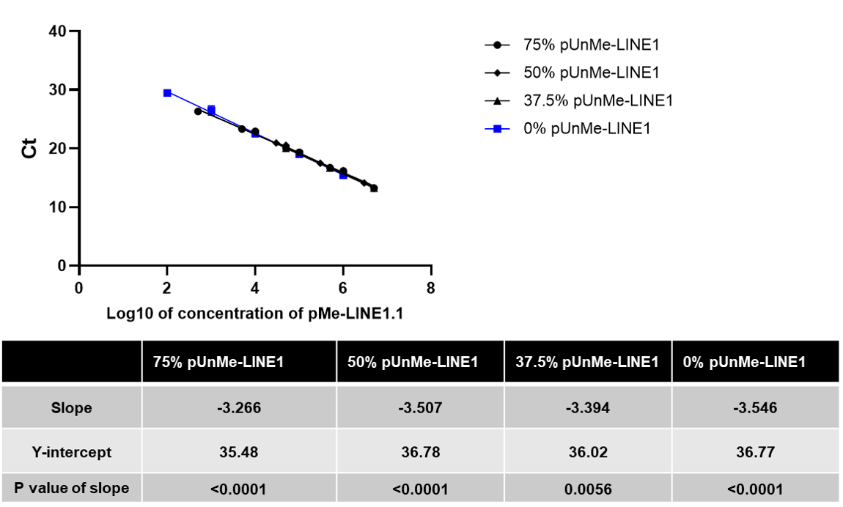


**S2 Fig. Plasmid pMe-LINE1.1 was mixed with plasmid pUnMe-LINE1 in different ratios.** Standard curves of pMe-LINE1.1 of each mix were built by using simple linear regression analysis. The different between the curves (Slope and Y-intercept) is not significant (p=0.1225 and p=0.9085 respectively). The pooled slope equals -3.409 and the pooled Y-intercept equals 36.19.
