# Supplementary material for: Standardization of DNA amount for bisulfite conversion for analyzing the methylation status of LINE-1 in lung cancer
Source: PLoS One. 2021 Aug 17;16(8):e0256254. doi: 10.1371/journal.pone.0256254 (PMC8370637; doi:10.1371/journal.pone.0256254)
Supplement: S3 Fig — Direct sequencing of the IC amplified from bisulfite-treated samples containing 106 IC copies mixed with 500 ng of DNA (A) or 108 IC copies mixed with either 500 ng (B) or 5 ng (C) of genomic DNA. Direct sequencing of the LINE-1 sequence amplified from bisulfite-treated samples containing 106 IC copies and 5 ng (D), 50 ng (E) and 500 ng (F) of genomic DNA. Unconverted cytosines were indicated by arrows. (DOCX) [file pone.0256254.s006.docx]

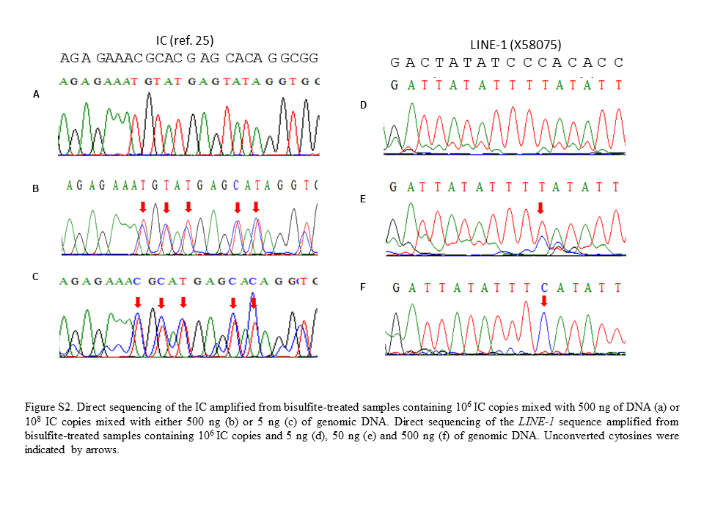


**S3 Fig.** **IC and *LINE-1* sequencing results**. Direct sequencing of the IC amplified from bisulfite-treated samples containing 10^6^ IC copies mixed with 500 ng of DNA (A) or 10^8^ IC copies mixed with either 500 ng (B) or 5 ng (C) of genomic DNA. Direct sequencing of the *LINE-1* sequence amplified from bisulfite-treated samples containing 10^6^ IC copies and 5 ng (D), 50 ng (E) and 500 ng (F) of genomic DNA. Unconverted cytosines were indicated by arrows.
