# Supplementary material for: Standardization of DNA amount for bisulfite conversion for analyzing the methylation status of LINE-1 in lung cancer
Source: PLoS One. 2021 Aug 17;16(8):e0256254. doi: 10.1371/journal.pone.0256254 (PMC8370637; doi:10.1371/journal.pone.0256254)
Supplement: S4 Fig — Methylation level between LINE-1.1 and LINE-1.2 in non-cancerous lung diseases-NC (A) and in lung cancer-LC (B). Methylation measurement was performed on 5 ng of DNA converted by bisulfite. (*) p < 0.05; (**) p < 0.01; (***) p < 0.001; (****) p < 0.0001. Welch’t test were used for analysis. (DOCX) [file pone.0256254.s007.docx]

**S4 Fig**. *LINE-1* methylation. Methylation level between *LINE-1.1* and *LINE-1.2* in non-cancerous lung diseases-NC (A) and in lung cancer-LC (B). Methylation measurement was performed on 5 ng of DNA converted by bisulfite. (*) p < 0.05; (**) p < 0.01; (***) p < 0.001; (****) p < 0.0001. Welch’t test were used for analysis.
