# Supplementary material for: Standardization of DNA amount for bisulfite conversion for analyzing the methylation status of LINE-1 in lung cancer
Source: PLoS One. 2021 Aug 17;16(8):e0256254. doi: 10.1371/journal.pone.0256254 (PMC8370637; doi:10.1371/journal.pone.0256254)
Supplement: S5 Fig — (A) Invariability of ΔCT value analysed by ANOVA statistical method when using qPCR templates as following: a serial concentration of the pRef-LINE-1 (read line) and a serial concentration of the pMe-LINE-1.1 (blue line). (B) Validation of ΔΔCT method. The amplification efficiency of the bisulfite converted LINE-1.1 and methylated LINE-1.1 targets was examined using qPCR. A 10% methylation LINE-1 calibrator was made by mixing pMe-LINE-1.1 with pRef-LINE-1 in ratio 1:10 and the serial dilution of the mixture was made, then amplified by methylation independent and methylation dependent primer sets, respectively. The ΔCT (CTMe-LINE-1.1-CTRef-LINE-1.1) was calculated for each dilution. The data was fit using least-squares linear regression analysis (N = 9). (DOCX) [file pone.0256254.s008.docx]

**A**

**B**

**S5 Fig. Validation of the reference for *LINE-1* methylation measurement.** (A) Invariability of ΔCT value analysed by ANOVA statistical method when using qPCR templates as following: a serial concentration of the pRef-LINE-1 (read line) and a serial concentration of the pMe-LINE-1.1 (blue line). (B) Validation of ΔΔCT method. The amplification efficiency of the bisulfite converted *LINE-1.1* and methylated *LINE-1.1* targets was examined using qPCR. A 10% methylation *LINE-1* calibrator was made by mixing pMe-LINE-1.1 with pRef-LINE-1 in ratio 1:10 and the serial dilution of the mixture was made, then amplified by methylation independent and methylation dependent primer sets, respectively. The ΔCT (CT_Me-LINE-1.1_-CT_Ref-LINE-1.1_) was calculated for each dilution. The data was fit using least-squares linear regression analysis (N = 9).
